# Transient Post-infectious Multisystem Inflammatory Overlap Presentation in a Pediatric Patient With Neurological, Hepatic, Gastrointestinal, and Autoimmune Manifestations: A Rare Case From Azerbaijan

**DOI:** 10.7759/cureus.116171

**Published:** 2026-09-13

**Authors:** Divya Ravikumar, Aliakbar Hasanov, Fakhriya Mammadova, Ahmed Roshan, Atique Anver

**Affiliations:** 1 Department of Pediatrics, Azerbaijan Medical University, Baku, AZE

**Keywords:** autoimmune seropositivity in children, hepatic and neurological overlap manifestations, pediatric multisystem inflammatory syndrome, pediatric neuroinflammatory disorders, post-infectious inflammatory overlap syndrome

## Abstract

Pediatric multisystem inflammatory overlap presentations represent a significant diagnostic dilemma. These transient, reactive conditions frequently mimic severe, chronic autoimmune, neoplastic, or demyelinating disorders, leading to potential diagnostic confusion.

Following an antecedent respiratory infection, post-infectious immune activation can manifest as a complex cluster of concurrent multi-organ findings, including incidental neuroinflammatory imaging changes, hepatic steatosis, gastrointestinal inflammation, and transient autoantibody positivity. Distinguishing these benign, self-limiting post-infectious responses from established systemic disease is challenging, as radiological and laboratory anomalies often appear disproportionate to the patient’s clinical status.

Recognizing transient post-infectious overlap phenotypes is essential to prevent overtreatment, unnecessary invasive interventions, and inappropriate long-term immunosuppressive therapy. A conservative management strategy supported by systematic, multidisciplinary monitoring and careful clinical correlation, rather than relying solely on isolated laboratory or imaging markers, ensures favorable clinical outcomes and confirms the benign nature of these syndromes. This case underscores that transient post-infectious inflammatory changes can closely mimic severe chronic pathology in pediatric populations. Systematic evaluation and cautious interpretation of isolated autoantibody positivity and incidental radiological findings are essential to prevent unnecessary invasive interventions and overtreatment while ensuring appropriate clinical monitoring.

## Introduction

Overlap syndromes in pediatric medicine refer to the coexistence of pathological or reactive findings involving multiple organ systems without a single clearly identifiable unifying diagnosis. Such presentations frequently raise concern for serious systemic illnesses including autoimmune disorders, connective tissue diseases, malignancies, central nervous system infections, and inflammatory demyelinating diseases [1]. In children, distinguishing transient reactive abnormalities from severe pathology is essential because radiological and laboratory findings may appear disproportionate to the patient’s clinical condition.

Positive autoimmune serological markers may further complicate clinical interpretation. Autoantibody positivity associated with conditions such as scleroderma and inflammatory myopathies may occur transiently following infectious or inflammatory processes and may not always indicate established systemic autoimmune disease [2]. Careful clinical correlation and longitudinal follow-up are therefore necessary before confirming chronic rheumatological diagnoses in pediatric patients.

Post-infectious inflammatory responses are established causes of transient neurological and systemic abnormalities in children. Beyond neuroinflammatory changes, such as small incidental gliotic foci, concurrent extra-neurological findings, including hepatic steatosis, gastrointestinal inflammation, and mild renal crystalluria, may further complicate the diagnostic evaluation and prompt extensive workups [3,4].

The term "overlap syndrome" is used in this report descriptively to denote concurrent multisystem inflammatory, neurological, hepatic, gastrointestinal, and autoimmune findings. These manifestations occurred in the absence of a fully established rheumatological or demyelinating disease entity.

We present a rare pediatric case characterized by simultaneous respiratory, neurological, hepatic, gastrointestinal, autoimmune, and metabolic findings that ultimately demonstrated a benign clinical course. This report provides valuable regional baseline clinical data from the South Caucasus, highlighting how systematic, conservative multidisciplinary management prevents unnecessary invasive procedures and unwarranted long-term immunosuppression in complex post-infectious overlap presentations.

## Case presentation

An 11-year-old male patient from Azerbaijan presented on day 0 with a nine-day history of fever and productive cough. There was no significant past medical history, chronic illness, autoimmune disorder, or prior neurological disease.

On admission, the patient appeared moderately ill. His body temperature was 37.5°C, oxygen saturation was 95% on room air, and respiratory rate was 24 breaths per minute. Physical examination revealed mild pallor, facial puffiness, and moist crackles on lung auscultation. No skin thickening, Raynaud phenomenon, proximal muscle weakness, Gottron papules, heliotrope rash, joint swelling, or vasculitic lesions were identified. 

Initial laboratory investigations demonstrated leukocytosis with a white blood cell count of 13.8 × 10⁹/L and markedly elevated inflammatory markers, including a C-reactive protein level of 77 mg/L. The erythrocyte sedimentation rate was within normal limits. Chest radiography demonstrated right lower lobe parenchymal infiltration consistent with acute pneumonia, and the patient was diagnosed with acute pneumonia (ICD-10: J18.8).

The patient received treatment with intravenous fluids, dexamethasone, and intravenous antibiotic therapy with ceftriaxone (50 mg/kg/day IV for seven days; brand name Bazocid), alongside supportive care. During hospitalization, he demonstrated marked clinical improvement. By day 5, follow-up investigations showed normalization of the white blood cell count and reduction of CRP to 1.69 mg/L. Repeat chest radiography demonstrated near-complete resolution of the pulmonary infiltrates.

Serial laboratory investigations demonstrated rapid normalization of inflammatory markers following treatment, supporting a favorable clinical response and reducing the likelihood of persistent systemic infection or progressive inflammatory disease. The trends in inflammatory markers during hospitalization and recovery are summarized in Table 1.

**Table 1:** Serial inflammatory marker trends during hospitalization CRP: C-reactive protein, WBC: white blood cell count, ESR: erythrocyte sedimentation rate.

Parameter	Day 0	Day 5	Reference range and units	Interpretation
CRP	77 mg/L	1.69 mg/L	<5mg/L	Strong recovery
WBC	13.8	7.22	4.5-11.0 × 10⁹/L	Normalization
ESR	Normal	15	0-20 mm/hr	Stable
Procalcitonin	-	0.11	<0.5 ng/mL	No sepsis

Further investigations were performed because of persistent systemic concerns and multisystem findings. Abdominal ultrasonography performed on day 7 revealed significant hepatic involvement. The liver was enlarged, measuring up to 174 mm, consistent with hepatomegaly. Diffuse increased hepatic echogenicity was observed, suggestive of grade II-III fatty liver infiltration (Figure 1). Despite these changes, the hepatic parenchyma remained homogeneous without evidence of focal lesions or masses. The intrahepatic and extrahepatic bile ducts were normal in caliber, with no evidence of biliary obstruction (Figure 2). Mild splenic enlargement was also noted, likely representing a reactive inflammatory change. Overall, the imaging findings were consistent with hepatomegaly associated with diffuse fatty liver disease without focal hepatic pathology.

**Figure 1:**
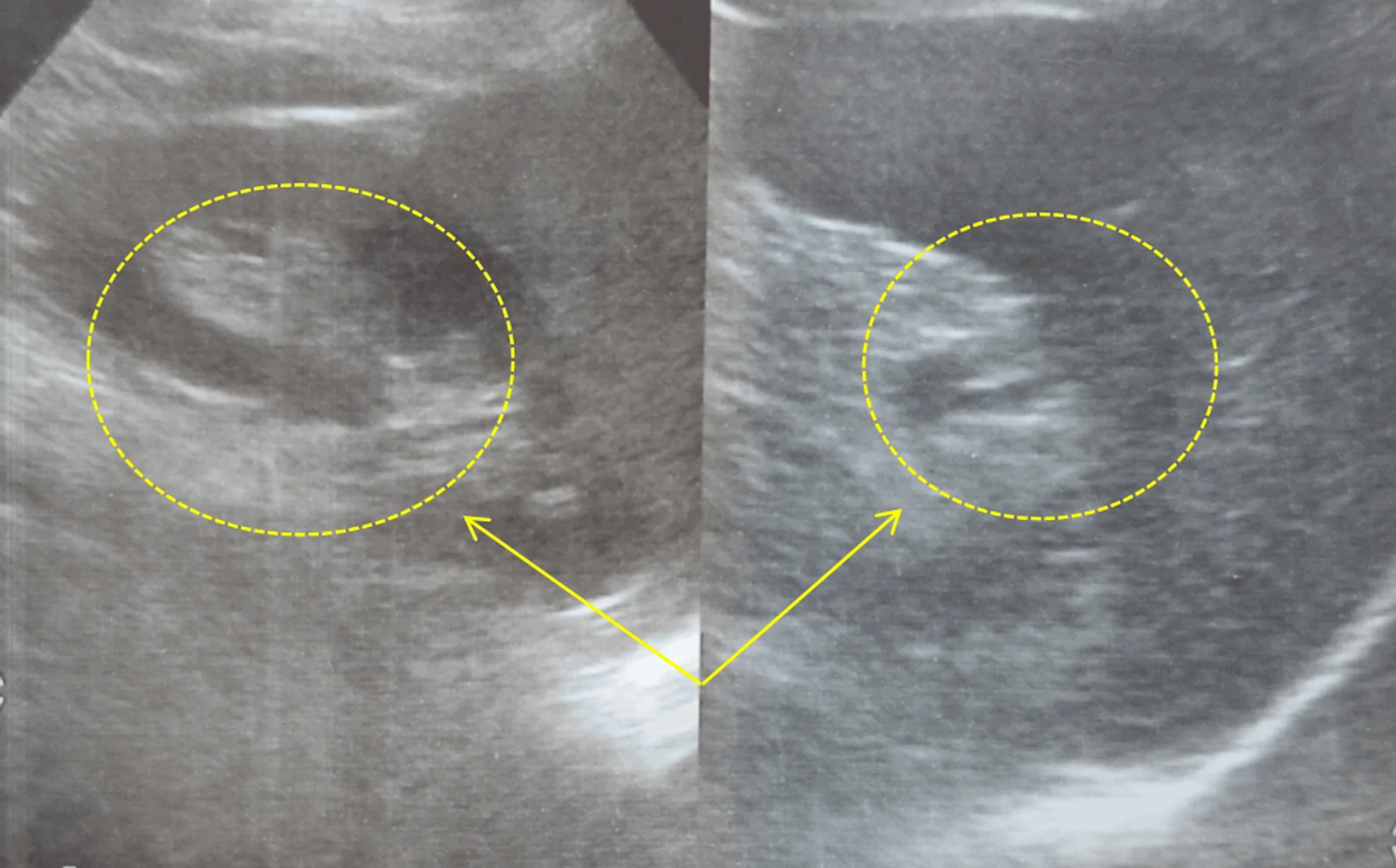
Abdominal ultrasound of the liver Transabdominal ultrasound of the right upper quadrant demonstrating significant hepatomegaly (174 mm) with diffuse increased hepatic parenchymal echogenicity, consistent with grade II-III fatty liver infiltration.

**Figure 2:**
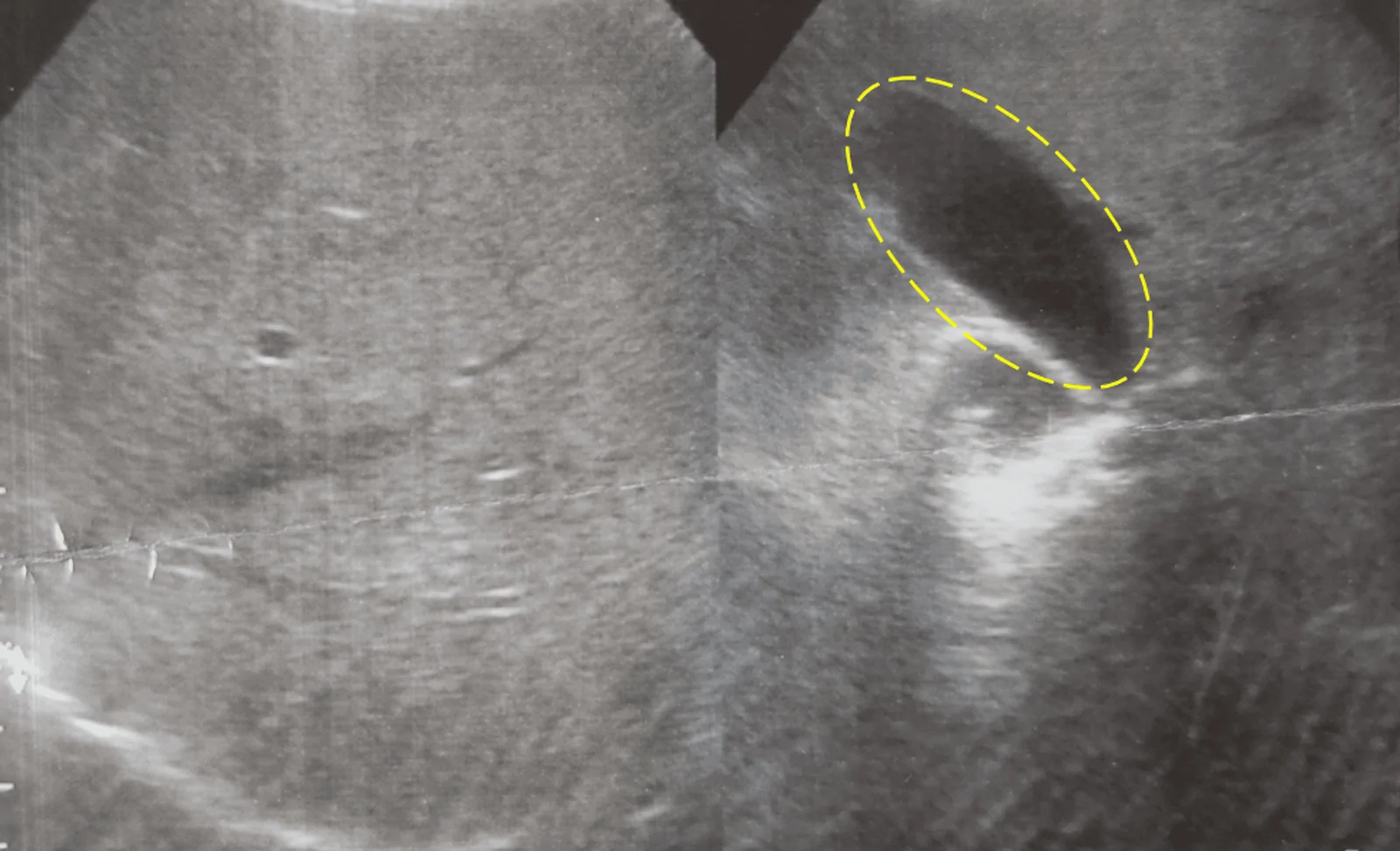
Abdominal ultrasound of the gallbladder and biliary tree Transabdominal ultrasound showing a moderately distended gallbladder with normal wall thickness and no gallstones or pericholecystic fluid. Intrahepatic and extrahepatic bile ducts are normal in caliber without evidence of biliary obstruction.

Given the multisystem involvement and persistent inflammatory concerns, an extended autoimmune and rheumatological evaluation was performed. Autoimmune panel testing demonstrated positivity for scleroderma- and myositis-associated antibodies, raising concern for a possible connective tissue overlap process. Autoimmune panel testing (line immunoassay) demonstrated positive titers for anti-Scl-70 (18 U/mL; reference range: <12 U/mL negative, 12-15 U/mL borderline, >15 U/mL positive) and anti-PM/Scl (22 U/mL; reference range: <12 U/mL negative), raising initial concern for a scleroderma/myositis overlap process. All other evaluated autoantibodies, including anti-Jo-1, anti-Ku, anti-Mi-2, anti-Ro52, anti-Sm, and ANA, were negative. However, the patient did not demonstrate clinical features suggestive of systemic sclerosis, inflammatory myopathy, juvenile dermatomyositis, or mixed connective tissue disease (MCTD). Specifically, there was no evidence of Raynaud phenomenon, skin thickening, digital ulcers, dysphagia, proximal muscle weakness, heliotrope rash, Gottron papules, arthritis, vasculitis, or progressive pulmonary involvement.

Additional laboratory evaluation demonstrated no evidence of persistent systemic inflammatory activation or progressive autoimmune disease. Muscle enzyme elevation was not identified, and inflammatory markers normalized during hospitalization. Rheumatological assessment favored transient post-infectious autoimmune activation rather than established connective tissue disease. Clinical and radiological follow-up demonstrated stability without progression of autoimmune manifestations.

Detailed neurological examination remained largely unremarkable throughout hospitalization and follow-up. Cranial nerve examination was normal, with no facial asymmetry, visual disturbance, or bulbar dysfunction identified. Motor examination demonstrated preserved muscle bulk, normal tone, and full power (Medical Research Council grade 5/5) in all extremities. Deep tendon reflexes were physiologic and symmetric, and plantar responses were flexor bilaterally. No sensory deficits, gait abnormalities, cerebellar signs, sphincter dysfunction, or coordination impairment were observed. The absence of objective neurological deficits was considered reassuring despite the radiological abnormalities detected on neuroimaging studies.

Brain magnetic resonance imaging (MRI) performed on day 10 demonstrated a punctate 3 mm FLAIR/T2 hyperintense gliotic focus within the left centrum semiovale. The lesion showed no associated diffusion restriction, contrast enhancement, surrounding edema, or mass effect. No additional supratentorial or infratentorial abnormalities were identified.

Spine magnetic resonance imaging performed on day 16 demonstrated mildly increased T2-weighted signal intensity involving the conus medullaris region without cord expansion or pathological enhancement, suggestive of a mild inflammatory or post-infectious process. Thoracic spine MRI performed on day 17 additionally demonstrated a Schmorl’s node with mild degenerative endplate changes but no compressive lesion or significant spinal canal compromise.

Upper gastrointestinal endoscopy performed on day 22 demonstrated antral erythematous gastritis, with active *Helicobacter pylori* (+++) infection confirmed via rapid urease testing and endoscopic gastric biopsy histopathology.

Additional investigations on day 23 confirmed persistent fatty liver changes. Procalcitonin levels remained low at 0.11 ng/mL, reducing the likelihood of systemic bacterial sepsis. Anti-streptolysin O titers were within normal limits, and Parvovirus B19 IgM serology was negative. Renal ultrasonography demonstrated structurally normal kidneys with small punctate hyperechoic micro-calculi measuring 2-3 mm without acoustic shadowing, suggestive of early nephrolithiasis. Mild intestinal gaseous distension with possible chronic colitis-related changes was also observed.

Repeat brain MRI performed on day 57 (week 8) demonstrated stability of the previously identified gliotic lesion without progression or new abnormalities. Abdominal radiographic imaging performed on the same date continued to demonstrate hepatomegaly.

Lumbar puncture performed on day 58 (week 8) demonstrated an opening pressure of 14 cm H2O. CSF analysis revealed a protein level of 0.28 g/L (reference: 0.15-0.45 g/L), glucose of 3.4 mmol/L (concurrent blood glucose: 5.5 mmol/L), and a nucleated cell count of 3 cells/µL (100% mature lymphomononuclear cells). CSF Gram stain, bacterial cultures, and multiplex viral PCR panel (including HSV-1/2, VZV, and enterovirus) were negative, and cytology confirmed the absence of malignant cells. These findings supported mild inflammatory or post-infectious neurological involvement without evidence of neoplastic or progressive demyelinating disease.

At the two-month clinical follow-up, the patient remained asymptomatic without recurrence of fever, respiratory symptoms, neurological deficits, muscular weakness, or dermatological manifestations. Repeat clinical assessment demonstrated no evidence of progression toward systemic autoimmune disease, inflammatory myopathy, demyelinating disorder, or chronic neurological pathology. The patient continued to demonstrate stable neurological imaging findings and favorable overall clinical recovery.

Comprehensive multidisciplinary evaluation excluded several serious differential diagnoses, including pediatric autoimmune connective tissue disease, central nervous system infection, progressive demyelinating disorders, and malignancy. Although autoimmune panel testing demonstrated positivity for scleroderma and myositis-associated antibodies, the patient did not demonstrate clinical or radiological progression consistent with systemic sclerosis, inflammatory myopathy, or MCTD.

Neuroimaging findings remained stable without progression, and cerebrospinal fluid cytology was negative for malignant cells. Inflammatory markers normalized rapidly following treatment, and the patient experienced complete clinical recovery without neurological deficits, muscular symptoms, or persistent systemic manifestations.

The multisystem findings were ultimately interpreted as transient post-infectious inflammatory and reactive changes with isolated autoimmune seropositivity and a benign clinical course.

## Discussion

This case represents a rare transient pediatric multisystem inflammatory overlap presentation involving the respiratory, neurological, hepatic, gastrointestinal, renal, and autoimmune systems following an acute respiratory infection. The coexistence of inflammatory neurological imaging findings, hepatomegaly with fatty liver infiltration, positive autoimmune serology, gastrointestinal inflammation, and elevated inflammatory markers initially raised concern for severe systemic disease, including connective tissue overlap syndromes, demyelinating disorders, malignancy, and central nervous system infection. However, the absence of progressive clinical deterioration, stable serial imaging, normalization of inflammatory markers, and favorable longitudinal follow-up ultimately supported a benign post-infectious inflammatory process rather than established chronic autoimmune or neurological disease.

One of the most notable aspects of this case was the positivity for scleroderma and myositis-associated antibodies during autoimmune evaluation. In pediatric patients, isolated autoimmune seropositivity may occur transiently following infectious or inflammatory illnesses and does not necessarily confirm established autoimmune disease [4,5]. Importantly, this patient lacked hallmark clinical manifestations of systemic sclerosis or inflammatory myopathy, including skin thickening, Raynaud phenomenon, proximal muscle weakness, dysphagia, characteristic dermatological findings, or progressive organ dysfunction.

A post-infectious inflammatory process likely explains the transient neurological abnormalities observed in this patient. The temporal relationship between pneumonia and the development of spinal cord signal changes and mild cerebrospinal fluid lymphomononuclear cells supports this hypothesis. Similar transient inflammatory changes have been described following respiratory infections in pediatric populations [1,4].

The solitary gliotic focus identified on brain MRI was non-enhancing and lacked associated edema or mass effect. Small incidental gliotic lesions are increasingly recognized in children and are often clinically insignificant. Importantly, no progression, focal neurological deficits, or evidence of demyelinating disease was identified during evaluation.

The hepatic findings were notable for hepatomegaly and grade II-III fatty infiltration. Pediatric fatty liver disease is increasingly recognized and may occur secondary to metabolic factors, transient inflammatory states, or infection-related hepatic stress [5]. The absence of biliary obstruction or progressive liver dysfunction supported a non-aggressive, reactive process rather than a progressive metabolic or primary hepatic disorder [4].

The gastrointestinal findings were consistent with active *H. pylori* gastritis, which may have contributed to the patient’s systemic inflammatory burden. The renal crystalluria observed was mild and clinically stable without evidence of significant renal pathology. *H. pylori*-associated gastritis may also have contributed to systemic immune activation in this patient. Chronic *H. pylori* infection has been associated with increased inflammatory cytokine production, immune dysregulation, and autoimmune activation in susceptible individuals. Although the exact contribution of H. pylori to the patient’s multisystem inflammatory presentation remains uncertain, chronic gastrointestinal inflammation may have amplified the overall systemic inflammatory burden.

Several differential diagnoses were considered during evaluation, including pediatric autoimmune connective tissue disease, central nervous system infection, acute demyelinating disorders, malignancy, and systemic inflammatory syndromes.

Several important differential diagnoses were considered during the evaluation of this patient because of the presence of multisystem involvement and neurological abnormalities. A central nervous system tumor was initially considered due to the presence of a gliotic lesion on brain MRI; however, the lesion demonstrated no contrast enhancement, mass effect, or interval progression on repeat imaging, making neoplastic pathology unlikely. Multiple sclerosis and other demyelinating disorders were also considered because of the neurological findings, but these were excluded due to the absence of dissemination in time and space, lack of recurrent neurological episodes, and stable radiological findings during follow-up.

Transverse myelitis was included in the differential diagnosis because of the increased T2 signal intensity within the conus medullaris region; however, the abnormalities were mild, non-progressive, and unaccompanied by significant motor, sensory, or autonomic dysfunction. Systemic sepsis was another important consideration given the markedly elevated inflammatory markers and initial respiratory infection, although the low procalcitonin level and rapid clinical improvement argued against severe systemic bacterial infection.

Autoimmune disease and connective tissue overlap syndromes were also considered because of the multisystem manifestations and positive autoimmune serology for scleroderma and myositis-associated antibodies [6,7]. Classic pediatric overlap syndromes, such as juvenile dermatomyositis, MCTD, systemic lupus erythematosus (SLE), and systemic sclerosis overlap, characteristically present with relentless organ involvement, systemic vasculitis, or fixed autoimmune pathology [1,8]. Nevertheless, the absence of definitive clinical features, progressive organ involvement, or persistent inflammatory activity reduced the likelihood of established systemic autoimmune disease. However, extensive investigations, including cerebrospinal fluid cytology, serial neuroimaging, autoimmune assessment, inflammatory markers, and clinical follow-up, did not support progressive systemic disease.

Given the patient’s extensive multisystem involvement, broad differential diagnoses were considered during the diagnostic evaluation. The coexistence of respiratory infection, neurological imaging abnormalities, hepatomegaly with fatty liver infiltration, gastrointestinal inflammation, elevated inflammatory markers, and positive autoimmune serology initially raised concern for several serious pathological conditions, including infectious, autoimmune, demyelinating, neoplastic, and systemic inflammatory disorders. Because many of these conditions may present with overlapping clinical and radiological findings in pediatric patients, a comprehensive multidisciplinary approach involving serial laboratory investigations, neuroimaging, cerebrospinal fluid analysis, autoimmune evaluation, and clinical follow-up was undertaken to exclude progressive or life-threatening disease processes. The major differential diagnoses considered in this case and the reasons for their exclusion are summarized in Table 2.

**Table 2:** Differential diagnoses considered in a pediatric multisystem inflammatory overlap presentation T2: transverse relaxation time, CSF: cerebrospinal fluid, MRI: magnetic resonance imaging, CNS: central nervous system, MOGAD: myelin oligodendrocyte glycoprotein antibody-associated disease, NMOSD: neuromyelitis optica spectrum disorder, CRP: C-reactive protein, B19 IgM: Parvovirus B19 immunoglobulin M.

CNS: differential diagnosis	Clinical features supporting diagnosis	Findings against diagnosis	Relevant investigations
Central nervous system infection (meningitis/encephalitis/myelitis)	Fever, elevated inflammatory markers, spinal cord T2 signal abnormality, mild CSF lymphomononuclear cells	No meningeal signs, no neurological deterioration, CSF negative for infectious organisms, normal procalcitonin, rapid clinical recovery	CSF cytology and analysis, brain MRI, spine MRI, procalcitonin
Acute disseminated encephalomyelitis (ADEM)	Post-infectious neurological imaging findings, possible inflammatory spinal cord lesion	Absence of encephalopathy, no multifocal neurological deficits, solitary non-specific gliotic lesion rather than diffuse demyelinating lesions, stable MRI findings	Brain MRI, spine MRI, neurological examination
Transverse myelitis	Increased T2 signal in conus medullaris region suggestive of inflammatory spinal cord involvement	No motor weakness, bowel/bladder dysfunction, sensory level, or progressive neurological deficits; imaging remained stable	Spine MRI, neurological assessment
Pediatric autoimmune/inflammatory disease	Multisystem involvement involving liver, CNS, and gastrointestinal tract	No characteristic autoimmune manifestations such as rash, arthritis, vasculitis, mucosal involvement, or persistent inflammatory markers; favorable spontaneous recovery	Clinical examination, inflammatory markers, serial monitoring
Malignancy (CNS tumor/leukemia/lymphoma)	Multisystem findings with hepatomegaly and CNS abnormalities initially raised concern	No mass lesion, no contrast enhancement, no cytological evidence of malignancy in CSF, normalization of inflammatory markers, stable imaging findings, no constitutional deterioration	Brain MRI, CSF cytology, abdominal imaging, serial laboratory tests
Demyelinating disorders (multiple sclerosis/MOGAD/NMOSD)	Gliotic brain lesion and spinal cord signal abnormality	Solitary small lesion without dissemination in time or space, absence of recurrent neurological episodes, stable repeat MRI, benign clinical course	Repeat brain MRI, spine MRI, clinical follow-up
Systemic bacterial sepsis	Fever, pneumonia, elevated CRP and leukocytosis	Low procalcitonin, absence of hemodynamic instability, rapid improvement with treatment, no evidence of multiorgan failure	Procalcitonin, blood investigations, clinical monitoring
Viral infection/post-viral syndrome	Fever, inflammatory findings, mild CSF lymphocytes, transient neurological abnormalities	Viral serology including Parvovirus B19 IgM negative; no persistent viral symptoms or progressive disease	Viral serology, CSF analysis
Non-alcoholic fatty liver disease (NAFLD)	Hepatomegaly, diffuse fatty infiltration on ultrasound	Young age and acute presentation suggested reactive or transient inflammatory component rather than isolated chronic metabolic disease	Abdominal ultrasonography, liver imaging
Metabolic or mitochondrial disorder	Multisystem involvement including hepatic and neurological findings	No developmental delay, chronic neurological symptoms, metabolic crises, or progressive systemic deterioration	Clinical assessment, imaging studies, follow-up
Incidental radiological findings with reactive systemic changes	Small isolated gliotic lesion, mild degenerative thoracic changes, transient inflammatory abnormalities	Supported by stable repeat imaging, absence of progression, and complete clinical recovery	Serial MRI imaging, clinical follow-up

To better contextualize the present case, a focused review of the recent literature published within the last five years was performed, including case reports, review articles, and original research studies related to pediatric multisystem overlap syndromes, autoimmune overlap conditions, post-infectious inflammatory disorders, and combined neurological-hepatic-gastrointestinal manifestations. The available literature demonstrates that pediatric overlap syndromes often present with heterogeneous clinical findings involving multiple organ systems, frequently mimicking severe autoimmune, infectious, demyelinating, or malignant conditions. Several studies have also highlighted the diagnostic complexity associated with transient autoimmune seropositivity, inflammatory neurological lesions, hepatic involvement, and systemic inflammatory responses in children. The following table summarizes the most relevant recent publications related to the clinical presentation and diagnostic considerations observed in the present case (Table 3).

**Table 3:** Recent literature on pediatric multisystem inflammatory and autoimmune overlap presentations relevant to the present case

Year	Article title	Article type	Key relevance to your case	Journal
2021	“Resolution of calcinosis using bisphosphonates in overlap syndrome – a case report”	Case report	Pediatric overlap syndrome involving juvenile dermatomyositis/systemic sclerosis overlap; discusses autoimmune overlap manifestations in children	BMC Rheumatology (SpringerLink) [8]
2021	“New onset of hepatic steatosis post-severe multisystem inflammatory syndrome in children (MIS-C): a case report”	Case report	Pediatric multisystem inflammatory disease with hepatic steatosis following inflammatory syndrome; relevant to fatty liver and systemic inflammatory response	International Journal of Environmental Research and Public Health (MDPI) [9]
2021	“Capillaroscopy as a diagnostic tool in the diagnosis of mixed connective tissue disease (MCTD): a case report”	Case report	Discusses juvenile mixed connective tissue disease with overlap features involving myositis and systemic sclerosis	BMC Rheumatology (Springer Link) [10]
2021	“Severe rhabdomyolysis in a child with multisystem inflammatory syndrome: an autoimmune mechanism?”	Case report	Pediatric inflammatory multisystem disease with autoimmune-mediated muscular involvement	Pediatric Neurology (PubMed) [11]
2021	“Pseudotumor cerebri syndrome as a neurologic involvement of multisystem inflammatory syndrome in children: a case report”	Case report	Demonstrates neurological manifestations in pediatric multisystem inflammatory syndrome	Journal of Tropical Pediatrics (PubMed) [12]
2021	“A case of overlap syndrome of systemic sclerosis and cryoglobulinemic vasculitis with central nervous system involvement”	Case report	CNS involvement in overlap autoimmune syndrome; relevant for neurological abnormalities and autoimmune overlap discussion	Journal of Rheumatic Diseases (PubMed) [13]
2021	“Severe onset of inflammatory myositis in a child: think to paraneoplastic myositis”	Case report	Pediatric inflammatory myositis with extensive systemic evaluation; useful for differential diagnosis discussion	Italian Journal of Pediatrics (Springer Link) [7]
2022	“Overlap syndrome in a 12-year-old girl with systemic lupus erythematosus and anti-OJ antibody-positive polymyositis: a case report”	Case report	Pediatric overlap syndrome involving lupus and polymyositis with positive autoimmune antibodies; highly relevant to positive myositis/scleroderma markers in your patient	Pediatric Rheumatology (Springer Link) [1]
2023	“The rheumatologic masquerader: a case report on SLE-dermatomyositis-systemic scleroderma overlap syndrome”	Case report	Adolescent overlap syndrome involving systemic sclerosis and myositis features; relevant autoimmune comparison	Rheumatology (OUP Academic) [14]
2024	“Microscopic polyangiitis in pediatric systemic lupus erythematosus: a unique presentation of pulmonary-renal syndrome and case report of an overlap syndrome”	Case report	Pediatric autoimmune overlap syndrome with multisystem involvement	CEN Case Reports (Springer Link) [15]
2025	“Overlapping connective tissue disease-polymyositis and diffuse systemic scleroderma: a case report”	Case report	Connective tissue overlap syndrome with polymyositis and systemic sclerosis overlap	Clinical Medicine Insights: Case Reports (Sage Journals) [16]
2026	“Overlap syndrome between autoimmune hepatitis and primary sclerosing cholangitis in a seven-year-old child: a case report”	Case report	Pediatric overlap syndrome involving hepatic autoimmune disease; useful for overlap syndrome discussion and hepatic manifestations	Cureus (Cureus) [17]

This case demonstrates the importance of cautious interpretation of isolated autoimmune markers and incidental radiological findings in pediatric patients. Over-investigation may expose children to unnecessary invasive procedures, prolonged immunosuppressive therapy, and psychological stress [18,19]. A systematic multidisciplinary approach allowed exclusion of severe pathology while supporting conservative management and close monitoring [20,21].

## Conclusions

This case describes a benign pediatric multisystem overlap syndrome characterized by acute pneumonia with complete recovery, transient neurological abnormalities, hepatomegaly with fatty liver changes, positive scleroderma- and myositis-associated autoimmune markers, *H. pylori*-associated gastritis, and mild renal crystalluria. Despite extensive multisystem and autoimmune findings, the patient experienced favorable clinical recovery without evidence of malignancy, chronic neurological disease, systemic sclerosis, inflammatory myopathy, or progressive autoimmune pathology.

This report highlights that multisystem findings and isolated autoimmune seropositivity in pediatric patients do not always indicate severe chronic disease. Post-infectious inflammatory changes may mimic serious neurological and rheumatological conditions, while incidental MRI abnormalities and transient autoimmune markers should be interpreted cautiously in children. Systematic evaluation remains essential to exclude life-threatening pathology while avoiding unnecessary invasive interventions and overtreatment. With appropriate supportive care and close monitoring, the prognosis in such overlap syndromes may be excellent.
